# Subcutaneous Chloral Injection for Puerperal Eclampsia

**Published:** 1893-01

**Authors:** 


SUBCUTANEOUS CHLORAL INJECTIONS IN PUERPERAL ECLAMPSIA. Deshages, of Orleans (*Abeille Medicin*, February 8, 1882), considers the subcutaneous injection of chloral a valuable method of its administration in puerperal eclampsia, where it is impossible to administer it by the mouth and there is a rectal intolerance. From his experience in seven different cases, Deshages believes it to be the safest working method of administering chloral. In all cases an initial dose of from 7 1-2 to 15 grains sufficed at once to quiet the patient and to lengthen the period between the seizures. The total quantity used was usually from 45 grains to 1 drachm, given either in three doses at intervals of 8 to 10 hours, or following a large initial dose, hourly, 1 1-2 to 3 grains. The largest quantity used was 75 grains. The effect of the large initial dose differs in individuals, according to the depth of the uremic intoxication, the condition of the os, the pains, to the nearness of the delivery. The injections are only to be used in comatose conditions, on account of the severe pain accompanying them, unless morphia or cocaine are added. The strength of the solution should not be greater than 1 : 10, and the syringeful should be very slowly injected.

Deshages has also used this method in the eclampsias of non-puerperal origin with good results.—*University Medical Magazine.*

